# Mouse Models of Aortic Pathologies: Deciphering Molecular and Cellular Mechanisms from Bench to Bedside

**DOI:** 10.1016/j.cjco.2026.06.010

**Published:** 2026-06-29

**Authors:** Alicia Baptista Vicente, Alexis Richard, Clara Gourhand, Céline Fassot, Laurent Loufrani

**Affiliations:** a University of Angers, Inserm U1083, CNRS 6015, MitoVasc unit, Team CarMe, SFR ICAT, F49000 Angers, France; b Division of Medical Genetics, Department of Internal Medicine, McGovern Medical School, The University of Texas Health Science Center at Houston, Houston, Texas, USA

**Keywords:** aortic aneurysm, aortic dissection, molecular mechanism, cellular mechanism, inflammation, animal model

## Abstract

**Background:**

Aortic pathologies, including abdominal aortic aneurysm, thoracic aortic aneurysm, and aortic dissection, are life-threatening conditions characterized by complex pathophysiology and a lack of simple, early, and reliable diagnostic tools.

**Methods:**

This review synthesizes current knowledge on the molecular and cellular mechanisms underlying aortic pathologies, with a particular focus on vascular smooth muscle cell dysfunction, extracellular matrix degradation, and inflammatory signaling pathways. The review critically evaluates the strengths and limitations of widely used murine models, including elastase infusion, calcium chloride application, angiotensin II infusion, and β-aminopropionitrile administration, in the context of current clinical knowledge.

**Results:**

Murine models provide valuable insights into the early stages of disease, which are often inaccessible in humans, and have been instrumental in elucidating key mechanisms involved in aneurysm formation and dissection. These models differ in their anatomic specificity, vascular remodelling dynamics, immune activation profiles, and translational relevance. Emerging complementary approaches, including advanced translational platforms and computational models, further improve the interpretation and translational value of preclinical findings.

**Conclusions:**

By linking experimental findings to clinical perspectives, this review proposes a bench-to-bedside framework for the study of aortic disease. Integrating complementary experimental models with human-relevant systems may improve mechanistic understanding and support future diagnostic and therapeutic strategies innovation in aortic aneurysm and dissection.

## Central Illustration

**Figure:**
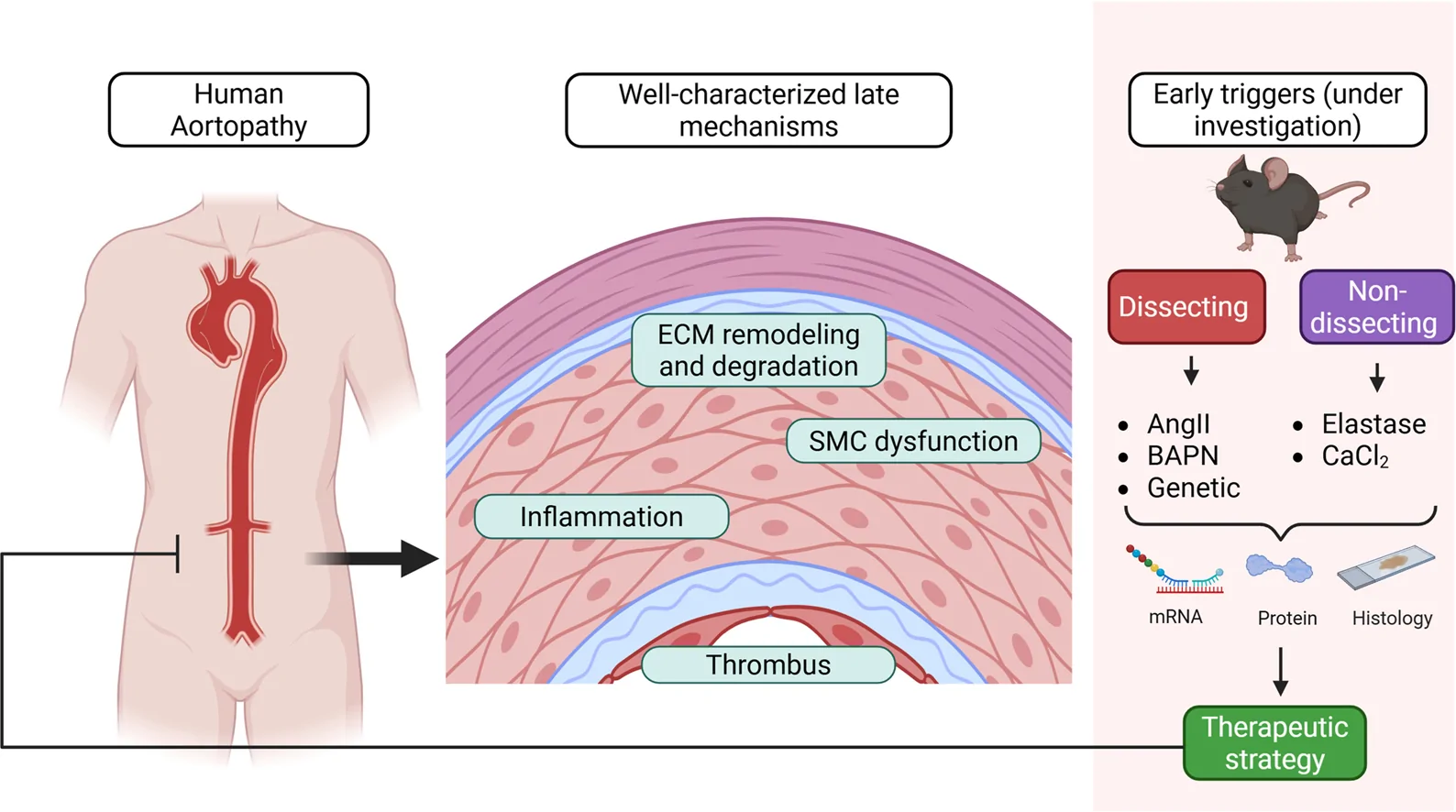

## Résumé

**Contexte:**

Les pathologies de l’aorte, notamment l’anévrisme de l’aorte abdominale (AAA), l’anévrisme de l’aorte thoracique (AAT) et la dissection aortique, sont des affections potentiellement mortelles caractérisées par une physiopathologie complexe et par l’absence d’outils diagnostiques simples, précoces et fiables.

**Méthodologie:**

Cette revue de littérature synthétise les connaissances actuelles sur les mécanismes moléculaires et cellulaires sous-jacents aux pathologies aortiques, en mettant particulièrement l’accent sur la dysfonction des cellules musculaires lisses vasculaires, la dégradation de la matrice extracellulaire et les voies de signalisation inflammatoires. À la lumière des connaissances cliniques actuelles, l’étude évalue de manière critique les atouts et les limites des modèles murins couramment utilisés, notamment la perfusion d’élastase, l’application de chlorure de calcium, la perfusion d’angiotensine II et l’administration de β-aminopropionitrile.

**Résultats:**

Les modèles murins fournissent des informations précieuses sur les stades précoces de la maladie, souvent inaccessibles chez l’humain, et ont joué un rôle déterminant dans l’élucidation des mécanismes clés impliqués dans la formation et la dissection des anévrismes. Ces modèles diffèrent par leur spécificité anatomique, la dynamique du remodelage vasculaire, les profils d’activation immunitaire et leur pertinence translationnelle. Des approches complémentaires émergentes, notamment des plateformes translationnelles avancées et des modèles computationnels, améliorent encore l’interprétation et la valeur translationnelle des résultats précliniques.

**Conclusions:**

En reliant les résultats expérimentaux aux perspectives cliniques, cette revue propose un cadre translationnel « du laboratoire au chevet du patient » pour l’étude des pathologies aortiques. L’intégration de modèles expérimentaux complémentaires à des systèmes pertinents pour l’humain pourrait améliorer la compréhension des mécanismes en jeu et soutenir l’innovation en matière de stratégies diagnostiques et thérapeutiques futures dans le domaine des anévrismes et des dissections de l’aorte.

Aortic aneurysms and dissections are life-threatening degenerative diseases of the aorta and represent a major cause of cardiovascular mortality in individuals aged over 55 years.^1^ Aneurysms are defined as localized arterial dilations exceeding 50% of the normal diameter, reflecting progressive structural weakening of the vessel wall (Figs. 1 and 2).^1^, ^2^, ^3^ They are characterized by extracellular matrix (ECM) degradation, medial thinning, and loss of vascular smooth muscle cells (VSMCs).^4^^,^^5^

**Figure 1:**
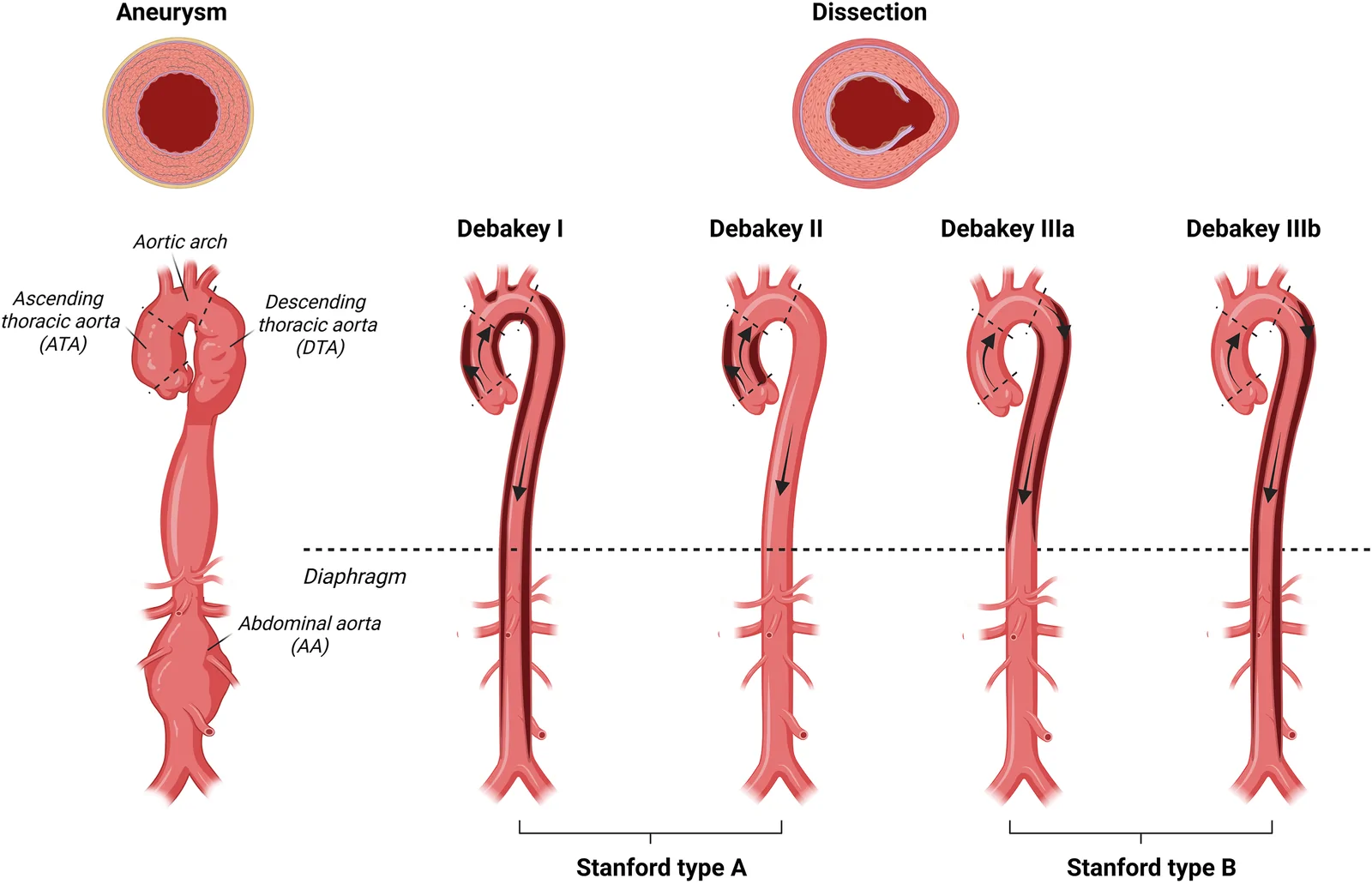
Anatomic localization and classification of aortic aneurysm and dissection. The aorta is divided into 3 main anatomic segments: the ascending thoracic aorta, the descending thoracic aorta, and the abdominal aorta, separated by the diaphragm. Aneurysms can occur in any of these segments and are broadly classified as thoracic or abdominal according to their anatomic location. Aortic dissections are classified according to the DeBakey (I–IIIb) and Stanford systems. DeBakey types I and II involve the ascending aorta, whereas type III affects the descending aorta, extending below the diaphragm in type IIIb. The Stanford classification distinguishes type A dissections involving the ascending aorta from type B dissections confined to the descending aorta.

Aneurysms may be fusiform or saccular and are classified according to their anatomic location. Approximately 75% arise in the infrarenal abdominal aorta (abdominal aortic aneurysms [AAAs]), whereas 25% involve the thoracic aorta (thoracic aortic aneurysms [TAAs]; Fig. 1).^6^^,^^7^

Despite some shared morphologic features, TAAs and AAAs differ substantially in anatomic context, risk factors, histopathology, genetic predisposition, and underlying molecular mechanisms.^2^^,^^7^^,^^8^ AAAs are predominantly associated with aging and cardiovascular risk factors, particularly smoking, hypertension, dyslipidemia, and chronic inflammation.^2^^,^^3^^,^^5^ Although frequently observed in patients with atherosclerosis, AAA pathogenesis is driven primarily by inflammatory infiltration, VSMC apoptosis, neovascularization, and progressive ECM degradation, thereby distinguishing it from classical occlusive atherosclerosis (Fig. 2).^3^^,^^5^ In contrast, TAAs are often linked to inherited connective tissue disorders, such as Marfan or Loeys-Dietz syndromes, or bicuspid aortic valve disease^6^^,^^9^, and they are characterized by early medial degeneration, elastic fiber disruption, impaired VSMC contractility, and dysregulated signalling pathways including transforming growth factor-beta (TGF-β).^4^^,^^8^ These alterations preferentially affect the ascending aorta and predispose individuals to acute complications, notably dissection and rupture.^1^^,^^7^

**Figure 2:**
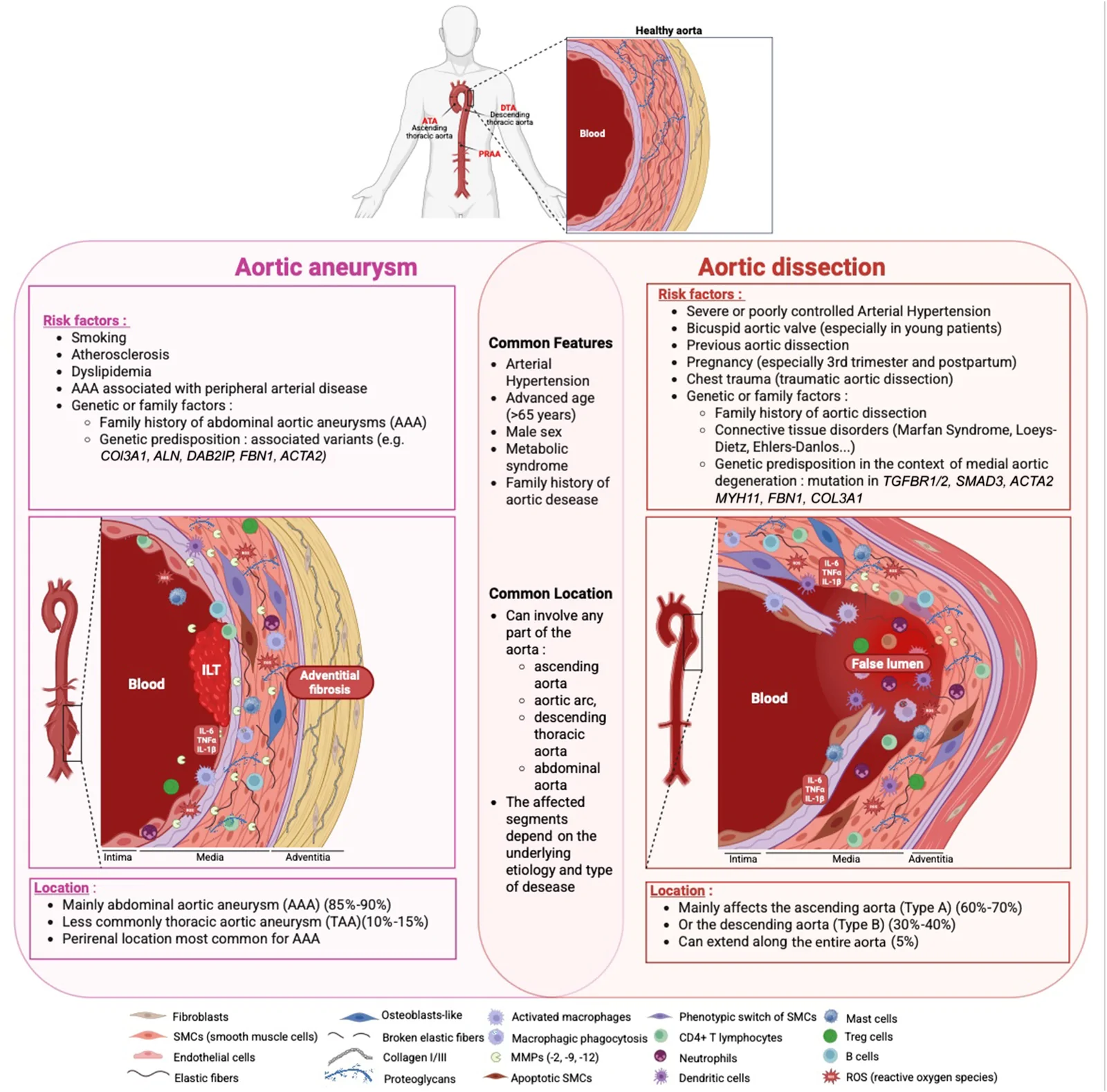
Comparative vascular remodelling in aortic pathologies. The figure provides a simplified overview of the main structural and cellular alterations observed in aortic aneurysm and dissection. The aneurysm panel primarily reflects features of abdominal aortic aneurysm (AAA), whereas the dissection panel illustrates key mechanisms associated with aortic dissection, particularly thoracic aortic dissection (TAD). The perirenal abdominal aorta (PRAA) is shown as a representative region commonly affected in experimental murine models. Major processes include vascular smooth muscle cell (VSMC) phenotypic switching, extracellular matrix degradation, inflammatory cell infiltration, and oxidative stress, all contributing to aortic wall weakening. Some risk factors, such as hypertension and genetic predisposition, are shared between aneurysm and dissection. IL, interleukin; ILT, intraluminal thrombus; MMP, matrix metalloproteinase; TNF, tumour necrosis factor.

Beyond chronic aneurysmal disease, the aorta also may be affected by acute aortic syndromes, among which aortic dissection (AD) is the most severe and prevalent. AD is initiated by an intimal tear that allows blood to enter the medial layer, generating a false lumen that can propagate longitudinally^10^ (Figs. 1 and 2). Dissections may occur *de novo* or complicate preexisting aneurysms and involve the ascending aorta in approximately two-thirds of cases,^10^ likely reflecting exposure to high systolic wall stress.^7^ Clinical classification relies on tear location and extent, with the Stanford system distinguishing type A dissections involving the ascending aorta from type B dissections confined to descending segments, and the DeBakey classification provides a more detailed anatomic framework useful for surgical decision-making (Fig. 1). Thoracic aortic dissection (TAD) involves predominantly the ascending aorta or descending thoracic segment and is strongly associated with medial degeneration, chronic hypertension, and heritable connective tissue disorders; it carries a high risk of acute rupture, aortic regurgitation, and malperfusion syndromes. In contrast, abdominal aortic dissection (AAD) is markedly rarer, usually affects an atherosclerotic and aneurysmal infrarenal aorta, and is more frequently linked to advanced age and diffuse vascular disease. TAD commonly presents with abrupt anterior chest or interscapular pain, whereas AAD more often manifests with abdominal, lumbar, or lower-limb ischemic symptoms due to iliac branch involvement. Management also differs—proximal TAD often requires emergent surgical or endovascular repair, whereas many stable infrarenal AAD cases may be managed initially with blood-pressure control and selective endovascular intervention.

Despite advances in molecular and genetic characterization, these conditions remain clinically challenging due to the lack of reliable biomarkers for early detection and risk stratification. Limited access to human aortic tissue, particularly at early disease stages, further constrains mechanistic investigation. Although AAAs, TAAs, and ADs differ in etiology and presentation, they share fundamental pathologic features, including ECM degradation, VSMC dysfunction, and maladaptive remodelling.^3^^,^^11^^,^^12^ In this context, murine models have become essential tools for dissecting disease mechanisms and translating experimental insights toward improved clinical management of aortic disease.^13^^,^^14^ Given these challenges, experimental models play a critical role in bridging mechanistic insights with clinical understanding.

This review provides an integrated overview of the cellular and molecular drivers of AAA, TAA, and AD, and critically evaluates the translational relevance of experimental models in advancing our understanding of human aortic disease (see Central Illustration).

**Central Illustration:**
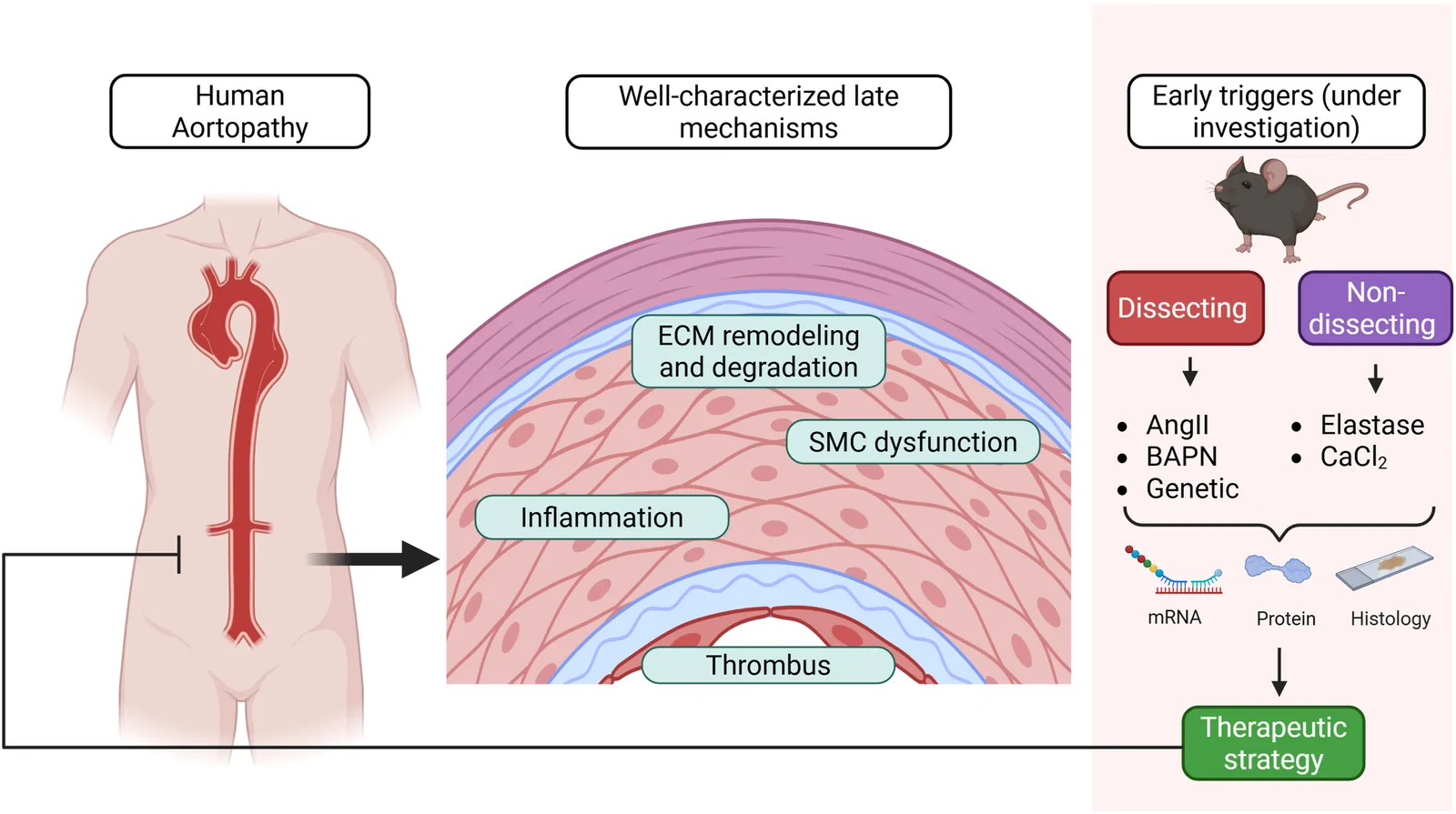
Molecular and cellular mechanisms of aortic aneurysm and dissection. This Central Illustration summarizes the key molecular and cellular mechanisms involved in aortic aneurysm and dissection, including vascular smooth muscle cell (SMC) dysfunction, extracellular matrix (ECM) remodelling, and inflammatory signaling pathways. It also highlights the contribution of experimental models in bridging fundamental research and clinical perspectives, supporting a translational “bench-to-bedside” approach. AngII, angiotensin II; BAPN, β-aminopropionitrile; mRNA, messenger RNA.

## Pathologic mechanisms of aortic aneurysm and dissection

Aortic aneurysms and dissections are clinically heterogeneous conditions with underlying mechanisms that vary depending on anatomic location and disease context (Fig. 2).^1^^,^^7^ These differences are further influenced by segment-specific properties, as thoracic and abdominal regions display distinct structural, embryological, and biomechanical characteristics that shape disease mechanisms. Despite these distinctions, all forms of aortic disease share core pathologic processes, including vascular smooth muscle cell apoptosis, ECM degradation, and immune activation.^4^^,^^8^^,^^11^^,^^12^ Together, these mechanisms contribute to medial weakening, loss of aortic wall integrity, and increased susceptibility to aneurysm progression and rupture.

This section provides an integrated overview of the key mechanisms underlying AAA, TAA, and AD, with a particular focus on how murine models have contributed to the identification and mechanistic validation of processes such as VSMC phenotypic switching, ECM degradation, and inflammatory activation.^13^, ^14^, ^15^

### Smooth muscle cell dysfunction

In aortic aneurysm and dissection, VSMCs undergo profound phenotypic and functional alterations that critically compromise medial integrity.^2^^,^^4^^,^^8^ Pathologic stimuli including mechanical stress, oxidative injury, inflammatory signalling, and genetic defects induce a shift from a contractile toward a synthetic, proliferative, and migratory phenotype.^8^^,^^12^^,^^16^ This phenotypic switch represents a central feature of abdominal and thoracic aneurysms as well as AD and has been documented consistently in human tissues and experimental models (Fig. 3; Table 1).^2^^,^^12^^,^^17^ Murine models, particularly AngII-induced and elastase-based models, have been instrumental in demonstrating the phenotypic switching of VSMCs from a contractile to a synthetic or inflammatory state, thereby contributing to aneurysm formation and progression.^15^^,^^17^

**Figure 3:**
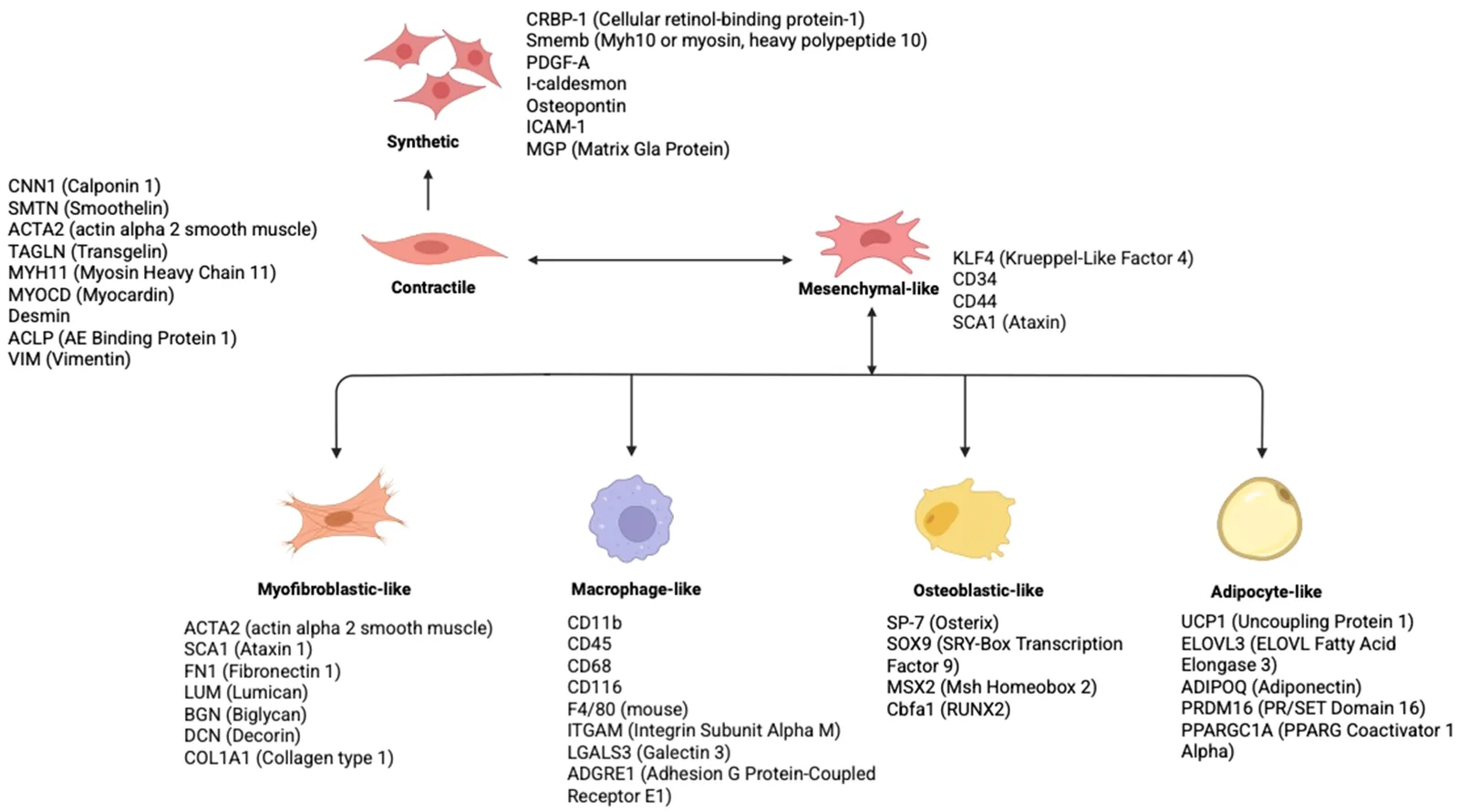
Schematic representation of vascular smooth muscle cell phenotypic transitions in aortic disease. The phenotypic transitions illustrated are supported by findings from widely used murine models, including AngII infusion and elastase-based models, as well as lineage-tracing and single-cell transcriptomic analyses. These models have been instrumental in demonstrating the transition of vascular smooth muscle cells (VSMCs) from a contractile phenotype toward synthetic, inflammatory, osteogenic-like, macrophage-like, and adipocyte-like states during aneurysm formation and progression. An important point to note is that these phenotypic states reflect functional and transcriptional reprogramming rather than true lineage conversion into distinct cell types. CD, cluster of differentiation; ICAM, intracellular adhesion molecule; PDGF, platelet-derived growth factor.

**Table 1:** Vascular smooth muscle cell phenotypes identified by single-cell RNA sequencing in human and murine aortic disease models

Species	Pathology model	Tissue	VSMC phenotype	References
Mouse : ApoE^−/−^ Myh11-CreERT2 mT/mGf/f Tgfbr2f/f	atherosclerotic plaque and aneurysm	ascending thoracic aorta	contractile, mesenchymal, fibroblast, macrophages-like, osteogenic, adipocyte-like	2,12,18
Mouse : Fbn1-C1041G/+	Marfan syndrome aortic aneurysm	aortic root and ascending thoracic aorta	contractile, fibroblast	9,24, 25, 26
Mouse : C57BL/6J	elastase-induced AAA (intraluminal or periadventitial elastase application)	infrarenal abdominal aorta	contractile, mesenchymal, macrophages-like	17,25,26
	CaCl_2_-induced AAA	infrarenal abdominal aorta	calcification-associated, pro-inflammatory and synthetic VSMC phenotype	13
	healthy aorta (Myh11-CreERt2/Confetti)	ascending and descending thoracic aorta	Ly6a/Sca1+ VSMC, with underregulated expression of contractile genes and overregulated expression of genes related to inflammation and growth factors	18
	elastase-induced AAA (7–14 d post-induction)	infrarenal abdominal aorta	contractile and quiescent, contractile and proliferative	17
Human	aortic aneurysm	ascending thoracic aorta	contractile, mesenchymal, fibroblast	2,12,18
Human (Fbn1c.7988G>A)	Marfan syndrome aortic aneurysm		contractile, fibroblast	9,22,23

This table summarizes VSMC phenotypic states identified across human and murine models of aortic aneurysm and dissection, including genetic and experimentally induced models (eg, elastase- and CaCl_2_-induced abdominal aortic aneurysm [AAA]). These studies highlight the phenotypic plasticity of vascular smooth muscle cells (VSMCs), characterized by transitions from a contractile state toward mesenchymal, inflammatory, osteogenic-like, and other modulated phenotypes, as revealed by lineage-tracing and single-cell transcriptomic analyses.

Transcriptional reprogramming drives this transition, notably through Krüppel-like factor 4 (KLF4), which suppresses contractile gene expression by inhibiting serum response factor binding to CArG box elements.^18^ Epigenetic mechanisms, including chromatin remodelling and histone modifications, further stabilize this altered state, particularly in response to oxidized lipids and platelet-derived growth factor signalling (Fig. 3).^8^^,^^18^ Single-cell transcriptomic analyses have revealed marked heterogeneity within diseased aortas, identifying transitional populations expressing mesenchymal, fibroblast-, chondrogenic-, or macrophage-like markers (eg, SRY-box transcription factor 9 [SOX9], galectin-3 [LGALS3], RUNX family transcription factor 2 [RUNX2]).^17^, ^18^, ^19^ These populations display enhanced matrix-degrading activity, reduced adhesive capacity, and pro-inflammatory behaviour, thereby promoting maladaptive remodelling.^11^^,^^12^

Beyond phenotypic modulation, medial degeneration is exacerbated by progressive VSMC loss through regulated cell death pathways.^4^^,^^8^^,^^20^ Increased apoptosis is a consistent feature of aneurysmal and dissected aortas and is driven by oxidative stress, inflammatory mediators, and mitochondrial dysfunction.^3^^,^^8^^,^^16^ Necroptosis, mediated by receptor-interacting serine/threonine-protein kinase 3 (RIPK3) and mixed lineage kinase domain-like pseudokinase (MLKL), has also been implicated, with pharmacologic inhibition attenuating inflammation and reducing rupture incidence in murine models.^21^ Although autophagy initially may exert protective effects, its dysregulation under chronic stress contributes to cell loss.^20^ In parallel, senescent smooth muscle cells accumulate with aging and sustained injury, adopting a senescence-associated secretory phenotype that amplifies inflammation and matrix degradation.^20^

Together, smooth muscle cell plasticity and depletion form a pathologic continuum that destabilizes the medial layer, accelerates ECM remodelling, and drives the progression of aneurysmal and dissecting aortic disease. Much of our current understanding of these mechanisms has been derived from experimental models, particularly murine systems, which allow controlled investigation of disease initiation and progression.

Non-coding RNAs (ncRNAs) have emerged as pivotal regulators of VSMC phenotype and survival, influencing aneurysm and dissection pathophysiology.^2^^,^^12^ They are typically classified by size (Fig. 4)—as small ncRNAs (< 200 nt), such as microRNAs (miRNAs), and long ncRNAs (> 200 nt). MiRNAs modulate gene expression post-transcriptionally by binding to 3′ untranslated regions of target messenger RNAs (mRNAs). The miR-143/145 cluster maintains the contractile VSMC phenotype^3^^,^^17^ whereas miR-21 and miR-29b regulate matrix remodelling, inflammation, and apoptosis; downregulation of miR-29b in murine AAA models correlates with increased matrix metalloproteinase expression and elastin degradation.^22^^,^^23^ Similarly, long non-coding RNAs (lncRNAs) have emerged as important regulators of VSMC proliferation and inflammatory signaling within aneurysmal tissue.^12^^,^^18^

**Figure 4:**
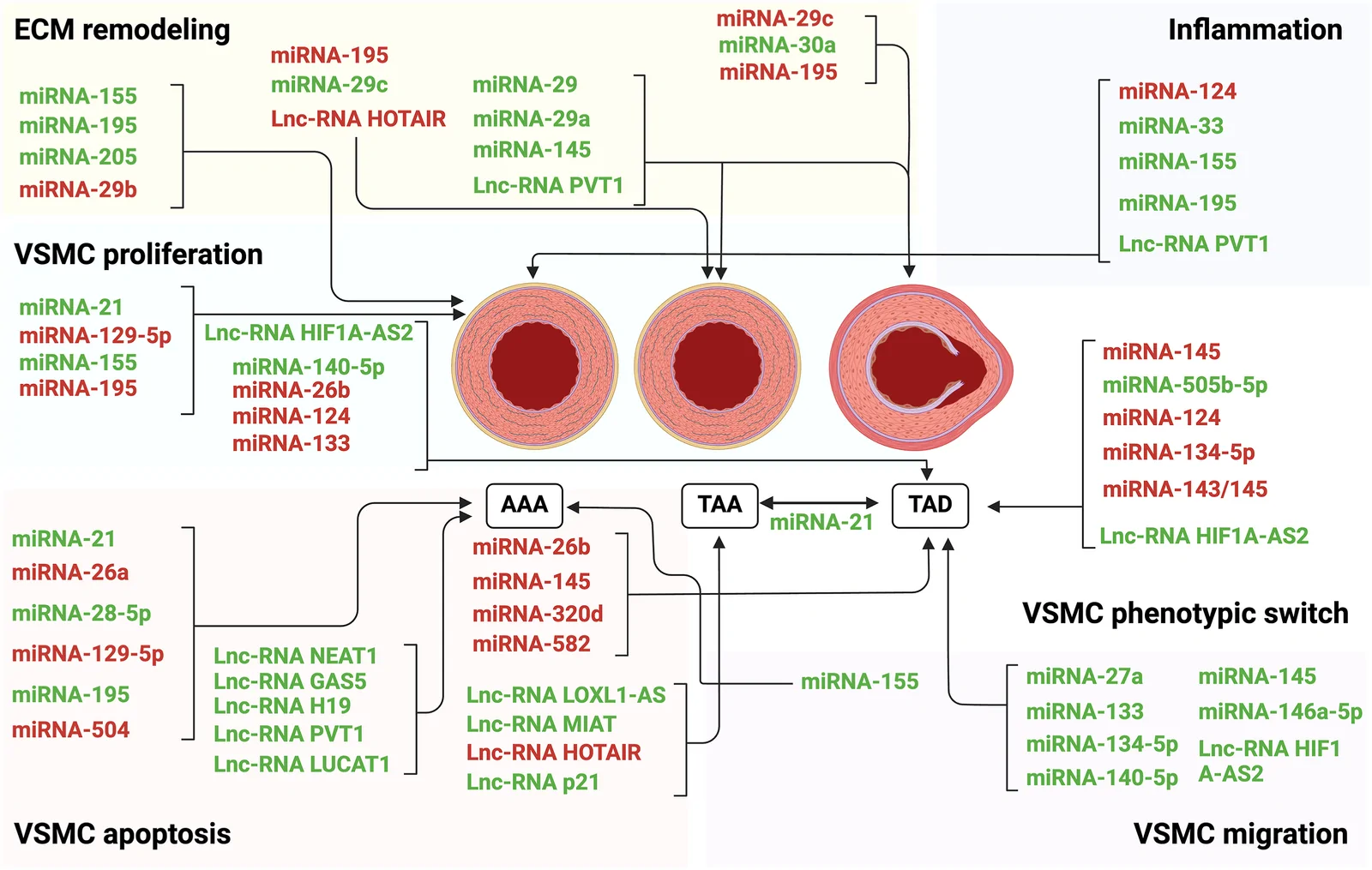
Non-coding RNA–mediated regulation of vascular smooth muscle cell (VSMC) functions in aortic aneurysm and dissection. This schematic summarizes the major microRNAs (miRNAs) and long non-coding RNAs (lncRNAs) involved in the regulation of key VSMC processes, including extracellular matrix (ECM) remodelling, proliferation, apoptosis, inflammation, migration, and phenotypic switching, in thoracic aortic aneurysm (TAA), abdominal aortic aneurysm (AAA), and thoracic aortic dissection (TAD). Upregulated molecules are shown in **green**, whereas downregulated molecules are indicated in **red**. These regulatory networks contribute to VSMC dysfunction and aortic wall remodelling underlying aortic disease progression.

Single-cell RNA sequencing from AngII-infused *ApoE*^*-/-*^ mice, elastase-perfused models, and human aortic specimens demonstrate the integration of these ncRNA-mediated pathways in VSMC plasticity and ECM regulation.^15^^,^^17^^,^^19^ Collectively, the interplay of miRNAs and lncRNAs orchestrates phenotypic switching, apoptosis, and matrix remodelling,^3^^,^^12^^,^^23^ suggesting that targeting ncRNA networks may offer novel therapeutic strategies for AAAs and TAAs as well as ADs.

### ECM remodelling and proteolytic regulation

AAs and ADs share a common feature of progressive ECM disruption, despite having differing locations, etiologies, and clinical courses.^3^^,^^8^ The aortic ECM, composed of elastin, collagen, microfibrils (fibrillins/fibulins), and proteoglycans (eg, versican, small leucine-rich proteoglycans [SLRPs]), provides tensile strength, elasticity, and mechanosensory cues to VSMCs. Loss of ECM integrity via genetic mutation, inflammation, or enzymatic degradation leads to VSMC phenotypic dysregulation, weakens the medial scaffold, and predisposes to dilation or dissection.^3^^,^^8^^,^^24^

Elastin forms durable elastic fibers through lysyl oxidase-mediated cross-linking and integration with microfibrillar scaffolds. Its degradation, observed in thoracic and abdominal AA, drives VSMC dedifferentiation and medial weakening.^3^^,^^24^ Microfibrils, composed of fibrillins and fibulins, act as structural scaffolds and mechanotransduction hubs; mutations such as *Fbn1* (Marfan syndrome) uncouple VSMCs from the ECM and accelerate aneurysm progression.^9^^,^^25^^,^^26^ Proteoglycans, including versican and aggrecan, regulate interlamellar cohesion, buffer mechanical stress, and sequester growth factors. Excess accumulation in TAA, AAA, or AD compromises medial integrity, amplifies inflammation, and contributes to cystic medial degeneration.^12^^,^^24^^,^^27^^,^^28^

ECM degradation is orchestrated by proteolytic systems (Table 2). Matrix metalloproteinases (MMP-2, MMP-9) degrade elastin and collagen, with activity regulated by tissue inhibitors of metalloproteinases (TIMPs). Dysregulation promotes medial thinning and wall fragility.^11^ A disintegrin and metalloproteinases (ADAMTS) proteases (notably ADAMTS-5 and -4) modulate proteoglycan turnover, with altered activity favouring aneurysm formation and dissection.^29^^,^^30^ Cysteine cathepsins (CatK, CatG, CatS) further degrade matrix components, and modulators such as osteoprotegerin and the kallikrein–kinin system amplify proteolysis and inflammation.^23^^,^^31^^,^^32^ Collectively, these pathways coordinate ECM remodelling, VSMC dysfunction, and aortic wall destabilization in aneurysmal and dissecting disease.^3^^,^^24^

**Table 2:** Major matrix metalloproteinases (MMPs) involved in extracellular matrix remodelling in aortic aneurysm and dissection

MMP	Main cellular sources	Class	Substrate	Stimulus	References
MMP-1	VSMCs, fibroblasts, leukocytes	Collagenase-1	collagens (I, II, III, VII, VIII, X), MMP-1, MMP-9, gelatin, proteoglycans	TNF-α, IL-1; requires plasmin or MMP-3 for activation	11
MMP-13	VSMCs	Collagenase-3	collagens (I, II, III, IV, IX, X, XIV), gelatin, MMP-9	TNFα,IL-1β	11
MMP-3	fibroblasts, epithelial cells, macrophages	Stromelysin-1	collagens (III, IV, V, IX, X), MMP-(1, 7, 8, 9, 13), fibronectin, gelatin, laminin, elastin	netrin-1	11
MMP-7	epithelial cells	Matrilysin	collagens (III, IV, V, IX, X, XI), proteoglycans, fibronectin, elastin, casein	plasmin	11
MMP-2	VSMCs, fibroblasts, macrophages	Gelatinase A	gelatin, collagens (I, IV, V, VII, X, XI, XIV), MMP-1, MMP-9, MMP-13, elastin, fibronectin, laminin	homocysteine, TNFα	11,12
MMP-9	VSMCs, fibroblasts, macrophages	Gelatinase B	gelatin, collagens (I, IV, V, VII, X, XI, XIV), elastin, fibronectin, plasminogen	homocysteine, TNFα, IL-1β	11,12
MMP-12	macrophages	Elastase	collagen IV, MMP-2, gelatin, elastin, fibronectin, casein, plasminogen, fibrinogen	IL-3	11
MMP-14	VSMCs, macrophages	Membrane type 1 MMP	collagens (I, II, III), MMP-2, gelatin, casein, elastin, vitronectin, fibronectin, laminin	not specified	11

This table summarizes key MMPs implicated in aortic wall remodelling across aortic pathologies, including abdominal aortic aneurysm, thoracic aortic aneurysm, and aortic dissection. It highlights their main cellular sources, functional classification, substrates, and regulatory stimuli. These proteases contribute to extracellular matrix degradation, elastin fragmentation, and vascular wall weakening, thereby playing a central role in aneurysm progression and dissection. IL, interleukin; TNF, tumour necrosis factor; VSMC, vascular smooth muscle cell.

Experimental murine models, including elastase-induced and AngII-based models, have been essential in identifying ECM degradation as a key driver of aortic wall weakening, particularly through protease activation and MMP upregulation.^15^^,^^23^^,^^33^

### Inflammation: a central amplifier of aortic disease

Inflammation serves as both a trigger and an amplifier of ECM degradation in AAA, TAA, and AD. This inflammatory response contributes directly to ECM degradation and medial weakening. Infiltrating immune cells, including macrophages, neutrophils, and T lymphocytes, release pro-inflammatory cytokines (interleukin {IL] 1β [, tumour necrosis factor [TNF]-α, IL-6),^2^^,^^12^ promoting MMP expression, VSMC apoptosis, and phenotypic switching. Neutrophil extracellular traps (NETs) have been shown to activate Hippo-YAP signalling, further exacerbating matrix degradation.^34^^,^^35^ Inflammasomes (NLR family pyrin domain containing 3 [NLRP3], absent in melanoma 2 [AIM2]) amplify cytokine maturation and immune recruitment, and adaptive immune cells (T helper 17 [Th17], interferon-gamma [IFN-γ] producers) intensify oxidative stress and medial degeneration.^2^^,^^12^

Even genetically driven TAA is further exacerbated by inflammatory mediators such as monocyte chemoattractant protein [MCP-1] and IL-6, as well as oxidative stress (eg, mediated by myeloperoxidase [MPO]), which enhance MMP activity and endothelial dysfunction. Mast cells and natural killer [NK] cells further modulate the immune microenvironment. In AD, tissue injury and intimal disruption lead to the release of damage-associated molecular patterns (DAMPs) that activate Toll-like receptor signalling, recruit leukocytes, and acutely compromise the ECM. Murine models, including AngII-infused *ApoE*^-/-^ mice and peri-adventitial elastase perfusion, have provided critical insights into the contribution of immune cell infiltration, including macrophages, particularly pro-inflammatory M1-like subsets, to chronic inflammation, ECM degradation, and vascular remodelling.^31^^,^^33^^,^^36^ Overall, these findings illustrate how murine models have been instrumental not only in reproducing key features of aortic disease, but also in establishing causal relationships between inflammation, ECM remodelling, and cellular dysfunction processes that remain difficult to investigate in human tissues.

## Mice Models of Aortic Aneurysm and Dissection

Animal models are essential tools for investigating the molecular and cellular mechanisms of aortic aneurysms and dissections and preclinical evaluation of therapeutic strategies.^2^^,^^31^ Among these, mouse models are particularly favoured due to their genetic manipulability, cost-effectiveness, short gestation, and suitability for high-throughput analyses.^31^^,^^32^ The diversity of available models permits targeted exploration of region- and mechanism-specific pathways.^31^ Murine models of aortic disease can be categorized according to the aortic segment affected (thoracic vs abdominal), the mechanism of induction (genetic, pharmacologic, or mechanical), and the resulting pathology (aneurysm vs dissection). Regarding pathology, murin models include non-dissecting aneurysms, characterized by progressive dilation without medial rupture, and dissecting aneurysms, involving medial delamination, intramural hematoma, or false lumen formation. This classification reflects both structural outcomes and underlying mechanisms, ranging from inflammation-driven ECM degradation in AAA to genetically or mechanically triggered dissection.^2^^,^^15^^,^^27^^,^^32^

Beyond pharmacologically induced models, genetic mouse models (eg, *Fbn1* and *Tgfbr* mutations) have been widely used to investigate thoracic aortic aneurysms and provide key insights into heritable forms of aortic disease.^9^^,^^26^ In addition to Marfan syndrome models, murine models of Loeys-Dietz syndrome, particularly involving mutations in TGFBR1 and TGFBR2, have provided important insights into dysregulated TGF-β signalling and its role in aortic aneurysm development.^37^ Combination models, such as β-aminopropionitrile (BAPN) administration combined with AngII infusion, can induce both thoracic and abdominal aortic pathologies and better reflect the multifactorial nature of human disease.^38^

Sex differences also have been reported in several murine models, with male mice generally exhibiting increased susceptibility to aneurysm formation and dissection, highlighting the importance of considering sex as a biological variable in experimental design.^39^ These differences may influence disease severity, incidence of dissection or rupture, and response to experimental interventions, thereby impacting the interpretation and reproducibility of experimental outcomes. As shown by Han et al, in a sex-specific prediction model and based on the UK Biobank, men may be predisposed to structural destruction potentially mediated by ECM degradation, whereas women may be predisposed to intrinsic failure potentially involving metabolic dysregulation and cell apoptosis.^40^

The most-used mouse model to study the pathogenesis of AAA is the angiotensin II infusion delivered via osmotic mini-pump for 28 days. Models also differ in their temporal dynamics and disease progression, with models such as elastase inducing rapid dissection or rupture, whereas others such as AngII (ApoE-/-) allow the study of progressive aneurysm development as shown in Table 3. For example, aortic dissection preceded altered flow patterns, and macrophage infiltration was a prominent characteristic of developing AAAs.^15^^,^^32^^,^^33^ Although no single model fully recapitulates human disease complexity, the diversity of available mouse models permits targeted exploration of region- and mechanism-specific pathways. However, important limitations remain, including species-specific differences in aortic structure and biomechanics, as well as the inability of individual models to fully recapitulate the complexity and chronic progression of human disease.

**Table 3:** Comparative overview of murine models of aortic aneurysm and dissection

Model	Segment	Induction	Timeline	Pathology	Strengths	Limitations	References
Elastase (PPE, intraluminal)	infrarenal abdominal aorta	intraluminal perfusion (surgical)	∼14 d	non-dissecting AAA	high reproducibility; strong inflammatory and proteolytic response	no ILT; limited rupture; invasive procedure	26,35,36
Elastase (periadventitial)	infrarenal abdominal aorta	periadventitial application	∼14 d	non-dissecting AAA	less invasive; reproducible; suitable for mechanistic studies	no ILT; rare rupture	26,36
CaCl_2_ model	infrarenal abdominal aorta	periadventitial chemical injury	2–6 wk	non-dissecting AAA with calcification	simple; models calcification and sterile inflammation	no ILT; limited clinical complexity	37, 38, 39, 41, 42
AngII (*ApoE*^*-/-*^*)*	suprarenal abdominal aorta	osmotic minipump infusion	2–4 wk	AAA with dissecting features (IMH)	mimics inflammation, oxidative stress, atherosclerosis	suprarenal location; variable rupture	33,31, 32, 37
AngII + high-fat diet (*ApoE*^*-/-*^ / *Ldlr*^*-/-*^*)*	suprarenal and thoracoabdominal	infusion + diet	2–4 wk (acute), longer remodelling	dissecting aneurysm (IMH, remodelling)	strong inflammatory and metabolic context	rapid progression; differs from human AAA (ILT absent)	14,15,35,37
BAPN	thoracic aorta	LOX inhibition (drinking water/chow)	4–12 wk	TAD	models ECM fragility; no genetic manipulation required	high variability; mortality; limited inflammation	36,37,42
BAPN + AngII	thoracic + abdominal	combined chemical + hemodynamic stress	2–6 wk	severe dissecting aneurysm (TAD + AAA)	recapitulates multifactorial disease; high incidence	high mortality; difficult standardization	34,36,42
Genetic models (*Fbn1*, *Tgfbr*)	thoracic aorta (ascending/root)	germline mutation	progressive (wk–mo)	TAA (heritable forms)	high clinical relevance (Marfan, LDS)	limited inflammation; model-specific phenotypes	9,24
Humanized mouse models	variable	engraftment of human cells (NSG/NOG)	variable	human-like immune/vascular responses	improved translational relevance; human-specific pathways	cost; technical complexity; incomplete immune maturation	45,46

This table provides a comparative overview of the principal murine models used to study aortic aneurysms and dissections. Models are categorized according to the aortic segment affected, induction mechanism, disease timeline, and resulting pathology. It highlights key differences in anatomic localization (thoracic vs abdominal), pathophysiological features (non-dissecting vs dissecting), and translational relevance to human aortic disease. The strengths and limitations of each model are summarized to facilitate appropriate model selection based on specific experimental objectives. AAA, abdominal aortic aneurysm; AngII, angiotensin II; BAPN, β-aminopropionitrile; ECM, extracellular matrix; ILT, intraluminal thrombus; IMH, intramural hematoma; LDS, Loeys–Dietz syndrome; PPE, porcine pancreatic elastase; TAA, thoracic aortic aneurysm; TAD, thoracic aortic dissection.

### Non-dissecting models

Non-dissecting models induce primarily AAAs, most commonly in the infrarenal aorta, and they are widely used to study progressive dilation without medial rupture. These non-dissecting models differ from thoracic aneurysm biology.

#### Elastase-induced abdominal aortic aneurysm in C57BL/6 mice

##### Intraluminal perfusion

The elastase-induced AAA model involves transient intraluminal perfusion of porcine pancreatic elastase (PPE) into a surgically isolated segment of the infrarenal aorta (Fig. 5).^23^^,^^31^^,^^32^ This enzymatic insult selectively degrades medial elastin fibers through proteolytic activity targeting elastic lamellae, leading to progressive dilation. Disease progression occurs in 2 phases—an early inflammatory phase (days 0-7), with recruitment of endothelial cells, VSMCs, fibroblasts, and neutrophils, followed by a late phase (by day 14), characterized by elastin fragmentation, collagen disorganization, macrophage and T cell infiltration, and upregulation of MMP-2 and MMP-9.

**Figure 5:**
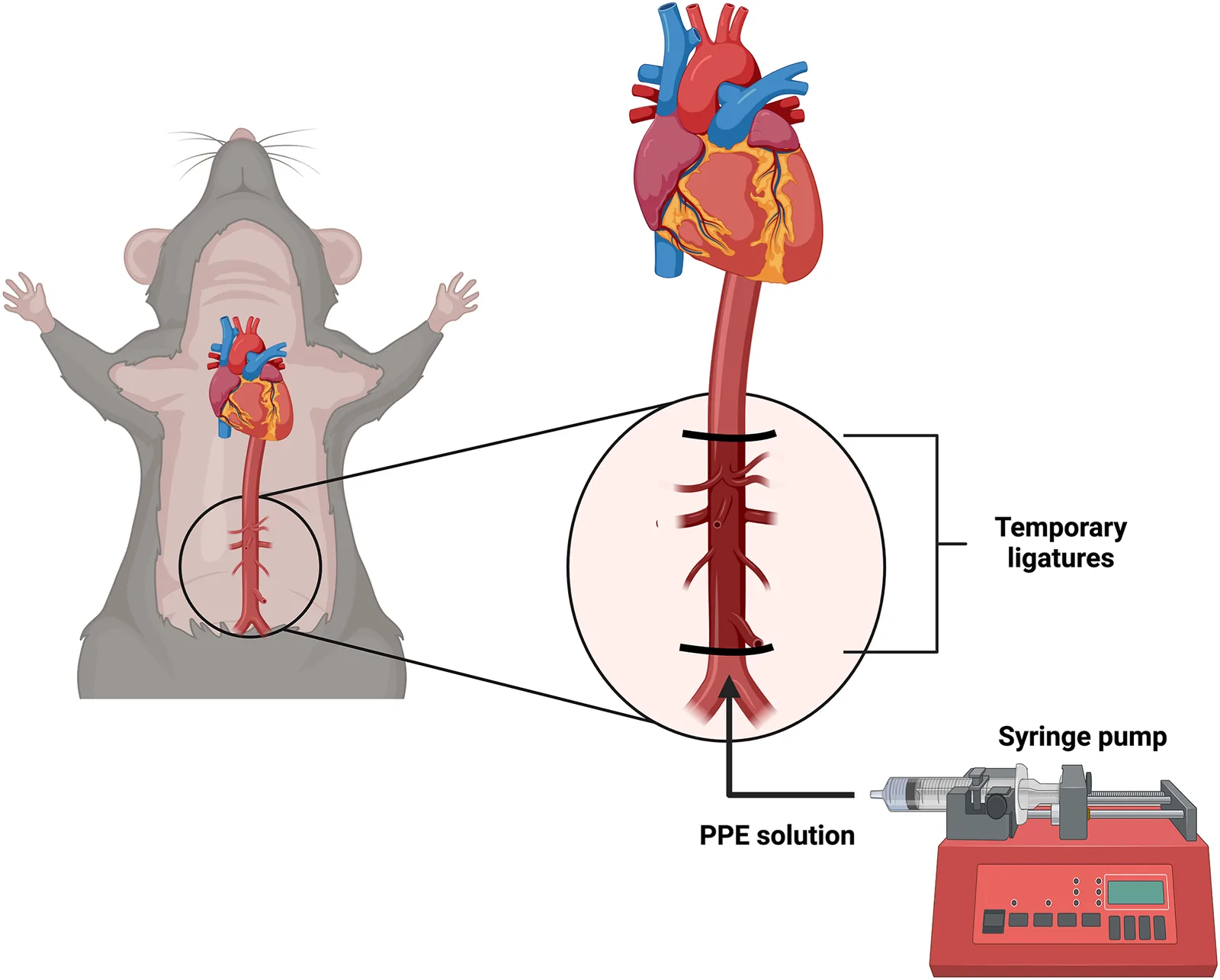
Elastase-induced abdominal aortic aneurysm (AAA) mouse model. Schematic representation of the intraluminal elastase perfusion model used to induce AAA in mice. The infrarenal abdominal aorta is surgically isolated between the left renal vein and the aorto-iliac bifurcation using temporary ligatures. Porcine pancreatic elastase (PPE) is then infused intraluminally via a syringe pump at controlled pressure. This enzymatic injury leads to degradation of medial elastic fibers, triggering inflammatory cell infiltration, extracellular matrix remodelling, and progressive aortic dilation. Aneurysm formation typically develops within 14 days following elastase perfusion.

The PPE model is highly reproducible and allows mechanistic dissection of inflammatory and proteolytic cascades driving AAA. The model’s main limitation is the absence of intraluminal thrombus (ILT) formation, restricting the study of thrombus-associated hypoxia, protease accumulation, and wall stress redistribution. Co-administration of β-aminopropionitrile (BAPN) and elastase in an AAA model does not affect the initial (2-week) dilation phase substantially, either mechanically or by altering the matrix content. Late mechanical and matrix effects of prolonged BAPN treatment are limited to the elastase-exposed segment of the aorta.^31^ This model affects primarily the infrarenal abdominal aorta, with aneurysm formation typically observed within 14 days, and it is widely used to study inflammation-driven ECM degradation, although it lacks key features such as intraluminal thrombus formation.^23^^,^^32^

##### Periadventitial application

A less invasive alternative to intraluminal perfusion applies PPE directly to the adventitial surface of the infrarenal aorta.^23^^,^^32^ This method induces reproducible aneurysms by day 14, with medial elastin degradation, inflammatory cell infiltration, and upregulation of MMPs.

The periadventitial model avoids luminal manipulation, reducing perioperative complications while preserving key pathologic features of AAA. Like the intraluminal model, formation of ILT and spontaneous rupture is limited. Its simplicity, reproducibility, and reduced technical demand make it well suited for mechanistic and interventional studies.^23^^,^^32^

#### Periadventitial application of calcium chloride (CaCl2) in C57Bl/6 mice

In this model, a 0.5 M CaCl_2_ solution is applied to the adventitial surface of the infrarenal aorta, followed by rinsing with phosphate-buffered saline.^37^ Calcium ions bind elastin^41^ and are internalized by VSMCs, leading to calcium phosphate crystal formation and hydroxyapatite deposition within the medial layer.^38^^,^^39^ These mineral deposits fragment elastic lamellae, stiffen the aortic wall, and generate a mechanical mismatch that promotes inflammation.

Elastin degradation products recruit monocytes, polarize macrophages toward a pro-inflammatory M1 phenotype, and stimulate cytokine secretion, including TNF-α. The CaCl_2_ model reproduces key features of AAA, such as elastin loss, VSMC apoptosis, vascular calcification, and proteolytic activity.^37^^,^^42^ However, the model does not develop intraluminal thrombus (ILT), advanced atherosclerosis, or spontaneous rupture, limiting its direct translational relevance. The model’s simplicity and reproducibility make it particularly useful for studying calcification-driven remodelling and sterile inflammation.

### Dissecting models

Dissecting aneurysms are characterized by sudden separation of aortic wall layers following an intimal tear, resulting in an intramural hematoma (IMH) that may progress to a false lumen and aortic dilation. These models are less commonly used than non-dissecting AAA models but are essential to the study of the pathophysiology of aortic dissection, including medial degeneration, elastic fiber disruption, and inflammatory responses.

Experimental models typically combine structural wall weakening with hemodynamic stress to reproducibly induce dissections in mice.^15^^,^^32^ Among these, AngII-based models are particularly important, as they frequently exhibit overlapping aneurysmal and dissecting features.^15^^,^^37^

#### Angiotensin II infusion in ApoE^-/-^ mice

Chronic subcutaneous infusion of AngII (500-1000 ng/kg/min for ∼28 days) via osmotic pumps in non-hypercholesterolemic *ApoE*^*-/-*^ mice (Fig. 6) induces aneurysmal dilation in the suprarenal abdominal aorta in 60%-80% of animals, depending on strain, sex, diet, and infusion duration.^31^^,^^32^^,^^37^ Lesions may include medial tearing and intramural hematoma formation, indicating overlap with dissecting aneurysm features.^15^^,^^37^ This model recapitulates key human AAA features, including upregulation of MMP-2 and MMP-9, oxidative stress, and chemokines such as MCP-1 and chemokine (C-C motif) ligand 2 [CCL2].^35^^,^^37^ (Its main advantage is the coexisting atherosclerotic environment, as high-fat diets exacerbate plaque formation.^31^ Limitations include variable rupture incidence and the suprarenal location, whereas human AAAs are usually infrarenal.^15^^,^^32^

**Figure 6:**
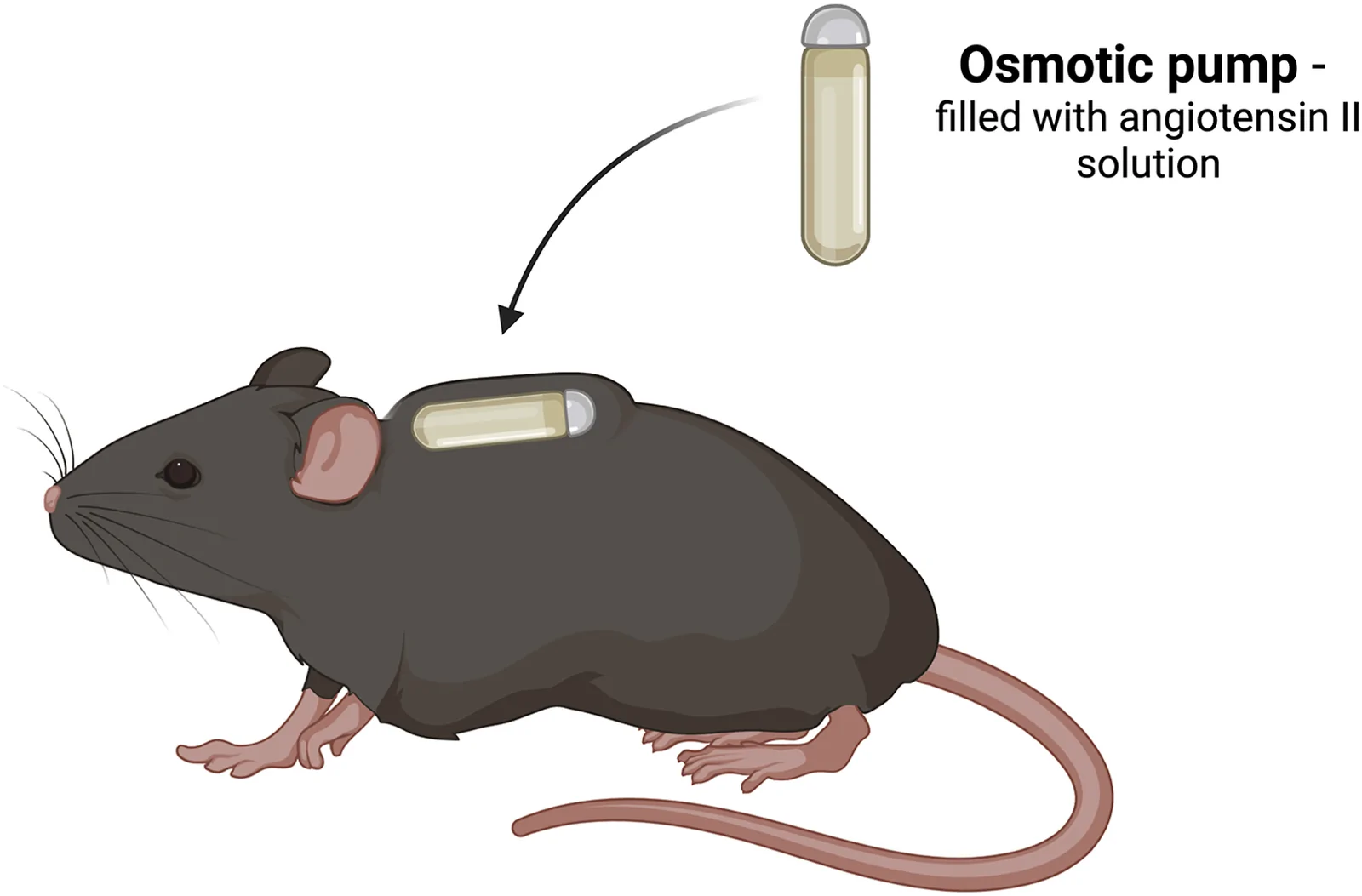
Angiotensin II–induced mouse model of aortic aneurysm and dissection. Schematic representation of chronic angiotensin II infusion via subcutaneous osmotic minipump in mice. Continuous angiotensin II delivery induces aortic pathology, predominantly in the suprarenal abdominal aorta, characterized by medial degeneration, inflammatory cell infiltration, oxidative stress, and extracellular matrix remodelling. This model frequently exhibits features of both aneurysm formation and aortic dissection, including medial tearing and intramural hematoma, and it is widely used to study the interactions among hypertension, inflammation, and vascular remodelling in aortic disease.

Refinements include intraluminal calcium phosphates (CaPO_4_) and the use of aged or diabetic mice, to better mimic human comorbidities.^32^ These variations aim to reproduce the systemic environment of aneurysmal disease. This model predominantly induces lesions in the suprarenal abdominal aorta, with rapid disease onset over days to weeks, and it is particularly relevant for studying the interaction among hypertension, inflammation, and atherosclerosis, although its anatomic location differs from that of most human AAAs.

#### Angiotensin II infusion with hypercholesterolemic diet: dissecting aneurysm and thoracoabdominal remodelling

##### Model overview

Chronic infusion of AngII in hypercholesterolemic mice (*ApoE*^*-/-*^ or *Ldlr*^*-/-*^*)* is a well established model of dissecting aortic aneurysms.^31^^,^^32^^,^^37^ This protocol induces suprarenal medial tears, IMH formation, aneurysmal dilation, macrophage infiltration, elastin fragmentation, and mural inflammation. Distinct features compared with human AAA include suprarenal localization, rapid expansion over days, and IMH rather than ILT.^14^^,^^15^ Extended infusion can lead to secondary remodelling, including partial thrombus resorption, ECM deposition, adventitial fibrosis, and sustained lesion growth (∼2.5 mm), influenced by genetic background and diet.^31^^,^^32^

##### Reconsidering the model

Recent evidence indicates that AngII-infused *ApoE*^*-/-*^ mice more closely model thoracoabdominal dissecting aneurysms than classical infrarenal AAA.^15^ In this model, medial tears often occur near branch points, IMH develops adventitially, and false lumen expansion is localized. Despite having anatomic differences from human AAA, the model captures key interactions among thrombus, branching architecture, and wall structural compromise, supporting its use for studying rupture-prone dissections. This model predominantly induces lesions in the suprarenal abdominal aorta, with rapid disease onset over days to weeks, and it is particularly relevant for studying the interaction among hypertension, inflammation, and atherosclerosis, although its anatomic location differs from that of most human AAAs.

### B-Aminopropionitrile (BAPN) model

Chemical agents can induce thoracic aortic dissection in experimental models. BAPN, an irreversible lysyl oxidase (LOX) inhibitor, destabilizes the aortic wall by preventing lysine cross-linking in collagen and elastin.^37^ It models matrix fragility observed in connective tissue disorders (eg, Ehlers–Danlos) and age or atherosclerosis-related degeneration.^32^ In mice, BAPN induces thoracic aortic dissection (TAD), particularly when combined with AngII, which adds hemodynamic stress to structural weakening.^36^^,^^43^ Key experimental variables include the following:

- genetic background: C57BL/6, FVB, or hybrids;
- age at initiation: 3-4 weeks vs 8-12 weeks;
- administration route: drinking water (0.1–1 g/kg/d), chow, or osmotic pump;
- duration: 4-12 weeks; and
- combination with AngII: administered concomitantly or sequentially, typically via minipumps.

These factors significantly modulate the incidence, location, and severity of aortic dissection, as well as the associated mortality rate.

This model reproduces TAD hallmarks, including elastic fiber fragmentation, medial delamination, adventitial hematoma, and increased susceptibility to dissection. The model allows targeted induction of thoracic dissection without genetic modification and reflects key aspects of human histopathology. However, strain selection and protocol standardization are critical, as small variations markedly affect lesion incidence, location, severity, and mortality.^32^^,^^43^

### Toward more human-relevant preclinical models in aortic disease research

Murine models have been pivotal for understanding aortic aneurysms and dissections (AAD), and they remain essential tools for mechanistic and preclinical studies. However, anatomic, physiological, and immunologic differences limit their translational relevance. Mice lack medial *vasa vasorum*, display species-specific hemodynamics, and show divergent immune responses, all of which can influence disease progression and therapeutic outcomes. In addition, key clinical features, such as intraluminal thrombus organization, complex atherosclerotic plaque dynamics, and long-term aneurysm evolution, are only partially recapitulated in murine systems. These limitations contribute to an incomplete representation of disease heterogeneity, particularly with respect to chronic progression, comorbidities, and long-term vascular remodelling.^12^^,^^14^^,^^31^

To overcome these limitations, complementary human-relevant experimental platforms have been developed. These include in vitro 3D tissue-engineered vascular models, induced pluripotent stem cell (iPSC)-derived VSMCs, and advanced biofabrication approaches such as bioprinting.^41^^,^^42^ In parallel, microphysiological systems and organ-on-chip technologies enable the study of vascular cell interactions under controlled hemodynamic conditions, thereby providing a more physiologically relevant environment.^44^, ^45^, ^46^, ^47^

In addition, recent advances in organoid-based systems and high-resolution digital analysis pipelines offer new opportunities to model vascular structure and function with increasing complexity.^48^ Integration of multi-omics data and computational approaches also contributes to a more comprehensive understanding of aortic disease mechanisms and may facilitate the identification of novel biomarkers and therapeutic targets.^49^, ^50^, ^51^

Together, these emerging platforms aim to bridge the gap between experimental models and clinical reality, improving biological fidelity and translational potential of preclinical research in aortic diseases.

#### Humanized mouse models

Humanized mice are generated by engrafting human cells or tissues into immunodeficient strains such as NOD-scid IL2rγnull (NSG) or NOG mice, which lack functional T, B, and NK cells. These models enable *in vivo* study of human-specific immune responses, cellular interactions, and gene regulation, thereby bridging conventional murine models and clinical studies.^46^^,^^47^ Humanization strategies include the engraftment of CD34^+^ hematopoietic stem cells to reconstitute a human immune system, vascular cells such as endothelial cells or VSMCs to model vessel-specific pathology, or peripheral blood mononuclear cells for short-term studies.^46^^,^^47^ Genetic humanization through targeted editing of disease-relevant genes may further improve relevance to heritable aortopathies. Nevertheless, these models remain limited by graft-vs-host disease, incomplete lineage maturation, and interspecies cytokine incompatibilities.^46^^,^^47^

In parallel, patient-derived primary cells and iPSC-based systems are central to precision modelling in aortic disease. These platforms retain patient-specific genetic and epigenetic signatures, enabling investigation of mutation-dependent phenotypes, therapeutic responses, and gene-editing strategies.^42^ When combined with 3D culture, organoid, or microphysiological platforms, these systems more faithfully reproduce aortic wall architecture and cellular crosstalk, thereby enhancing disease modelling, mechanistic understanding, and personalized therapeutic testing.^41^, ^42^, ^43^, ^44^, ^45^, ^46^, ^47^, ^48^

#### Vascular organoids and 3D bioprinting

Human iPSC–derived vascular organoids enable *in vitro* modelling of human aortic biology by recapitulating major vascular cell types, including endothelial cells, VSMCs, and fibroblasts, together with ECM remodelling, inflammatory signalling, and drug responses.^42^ Three-dimensional bioprinting further allows the fabrication of patient-specific vascular constructs with controlled architecture and perfusable structures, thereby expanding opportunities for disease modelling and translational testing.^41^

#### Comparative transcriptomics and bioinformatics

Comparative transcriptomic analyses are increasingly used to evaluate the translational relevance of preclinical aortic disease models. By aligning murine gene expression profiles with human aneurysm or dissection datasets, conserved molecular pathways and species-specific divergences can be identified.^43^ Comparisons of BAPN-induced murine dissections with human thoracic aortic dissection samples have revealed shared inflammatory and immune pathways. Integrative bioinformatics approaches combining pathway enrichment, network analysis, and machine learning now allow refined model evaluation and biomarker discovery.^49^, ^50^, ^51^

#### Computational and *in silico* models

Computational and *in silico* platforms have emerged as indispensable tools for modelling the complex biomechanics, genetics, signalling networks, and disease trajectories underlying AAD. Finite element analysis and fluid–structure interaction models allow patient-specific simulations of wall stress, flow dynamics, and rupture risk using imaging-derived geometries.^45^ Systems-level and multi-omics computational frameworks can further support disease stratification, biomarker discovery, and translational interpretation.^49^, ^50^, ^51^ These *in silico* tools provide scalable and complementary platforms alongside experimental models.

#### Microphysiological systems and organ-on-Chip platforms

Integration of organoids with microfluidic platforms supports dynamic perfusion, long-term culture, and real-time functional readouts.^48^ These platforms offer advanced tools for disease modelling and precision therapeutics, especially when combined with patient-derived iPSCs. Microphysiological systems integrate human vascular cells within microfluidic devices that reproduce physiologically relevant forces such as shear stress, cyclic strain, and pulsatile pressure.^44^, ^45^, ^46^, ^47^ “Aorta-on-chip” systems enable real-time assessment of endothelial dysfunction, ECM remodelling, and inflammatory responses under controlled mechanical conditions. Using patient-derived cells, these platforms facilitate personalized disease modelling and preclinical drug testing with high translational relevance.^44^, ^45^, ^46^, ^47^

#### Integration of multi-omics and machine learning

Integrating multi-omics datasets with machine learning has advanced systems-level studies of AAD. These approaches support disease subtyping, biomarker prioritization, and longitudinal modelling of disease progression.^49^, ^50^, ^51^ Coupled with increasingly interpretable computational frameworks, they improve alignment of preclinical models with patient-specific molecular landscapes and offer a scalable strategy for translating complex molecular data into mechanistic and therapeutic insights.

### Standardization and reporting: the 2025 guidelines framework

The growing heterogeneity of experimental models in aortic disease research has underscored the need for harmonized standards. Recent expert consensus recommendations, including ARRIVE 2.0 guidelines and field-specific proposals, provide a structured framework to enhance reproducibility, methodological rigour, and translational relevance in preclinical aortopathy studies.^31^ These recommendations emphasize appropriate model selection, transparent reporting of biological and environmental variables (eg, strain, sex, age, diet), and standardized outcome measures, including aortic dimensions, histopathologic scoring, elastin integrity, and biomechanical parameters. They further stress robust experimental design, including randomization, blinding, and comprehensive methodological disclosure. By promoting standardization, data sharing, and open science practices, this framework aims to ensure that preclinical models serve as reliable translational bridges rather than weak surrogates. Furthermore, Mohammadmoradi and Whiteheart compare major experimental models of abdominal and thoracic aortic disease, highlight methodological considerations, and propose standardized approaches to improve translational research, with particular focus on platelet–thrombus biology and glycoprotein VI (GPVI) as a potential biomarker and therapeutic target. Finally, they offer a decision grid and rigour checklist to harmonize model use, enhance reproducibility, and accelerate translation.^52^

### Clinical implications and therapeutic perspectives

Despite the substantial progress that has been made in experimental modelling, its translation into clinical practice remains limited, highlighting the need for more predictive and translationally relevant models.^31^ Preclinical studies have identified key mechanisms underlying wall degeneration, inflammation, and rupture; however, clinical applicability is hindered by disease heterogeneity and the absence of robust metrics.

#### Early diagnosis and biomarker development

AADs are often clinically silent until rupture, underscoring the need for early diagnostic biomarkers.^3^^,^^6^ Circulating proteases such as MMP-2, MMP-9, and cathepsins, as well as regulatory non-coding RNAs such as miR-29b, have been associated with aneurysm progression in murine models and human tissues.^11^^,^^22^ Integrative multi-omics strategies coupled with machine learning approaches are increasingly being used to improve risk stratification and longitudinal disease monitoring.^49^, ^50^, ^51^

#### Therapeutic perspectives

Several pharmacologic strategies show efficacy in experimental AAD models. Broad MMP inhibition, such as by doxycycline, limits aneurysm growth and preserves ECM integrity.^11^ Targeting inflammatory pathways also attenuates vascular inflammation and medial degeneration. More recently, modulation of regulated cell death pathways, such as necroptosis via MLKL inhibition, has demonstrated protective effects on VSMC survival and reduced rupture susceptibility.^21^ In parallel, epigenetic regulation mediated by non-coding RNAs has emerged as a key modulator of vascular remodelling and disease progression.^22^,^26^ Although they are largely preclinical, these approaches provide a mechanistic rationale for translational investigation.

In parallel, current clinical management of thoracic aortic aneurysms is guided by international recommendations, including recent American Heart Association (AHA) and European Society of Cardiology (ESC) guidelines, which emphasize imaging surveillance, strict blood pressure control, and timely surgical intervention based on aortic diameter and genetic background.^53^^,^^54^

### Toward precision and prevention

Advanced translational platforms, including patient-derived iPSCs, vascular organoids, and microphysiological systems, enable more personalized modelling of AAD and support patient-specific preclinical drug testing.^41^, ^42^, ^43^, ^44^, ^45^, ^46^, ^47^ By integrating patient-specific genetic backgrounds with physiologically relevant biomechanical cues, these approaches complement animal models and improve translational fidelity.^31^^,^^44^, ^45^, ^46^, ^47^

From a public health perspective, AAA remains strongly associated with modifiable risk factors such as smoking, hypertension, and dyslipidemia.^2^^,^^3^^,^^6^ Population-based screening, particularly in older men, combined with optimized cardiovascular risk management, remains among the most effective strategies to reduce disease-related mortality. In contrast, thoracic aortic aneurysms are frequently linked to genetic predispositions. Genetic screening is therefore increasingly integrated into clinical management, particularly in patients with a family history or suspected heritable aortopathies. Identification of pathogenic variants in genes such as *Fbn1*, *Tgfbr1*, or *Tgfbr2* enables early diagnosis, refined risk stratification, and personalized surveillance.^9^^,^^37^

Important differences between genetic and non-genetic aortic diseases, in addition to their etiology, are their underlying biological mechanisms and disease trajectories. Heritable thoracic aortopathies are primarily driven by ECM alterations and dysregulated TGF-β signalling, whereas non-genetic forms, particularly AAA, are more strongly associated with inflammation, oxidative stress, and environmental risk factors.^3^^,^^8^ These distinctions also have important implications for experimental modelling, as genetic models (eg, *Fbn1* or *Tgfbr* mutations) primarily recapitulate heritable thoracic aortopathies, whereas pharmacologic models more closely recapitulate inflammation-driven abdominal aortic aneurysms. In particular, models of Loeys–Dietz syndrome, involving *Tgfbr1* or *Tgfbr2* mutations, provide complementary insights into TGF-β–driven vascular pathology and further highlight the heterogeneity of thoracic aortic disease.

Future studies integrating multi-scale modelling, patient-derived systems, and longitudinal clinical data are essential to enhance the predictive value of preclinical findings and accelerate the transition toward precision-based prevention strategies in aortic disease.^45^^,^^49^, ^50^, ^51^

## Conclusion

Over the past 2 decades, research on aortic aneurysms and dissections has advanced substantially through progress in molecular biology, experimental modelling, and translational approaches. Murine models have been instrumental in uncovering pathogenic mechanisms, but their anatomic and physiological divergence from human disease limits direct clinical extrapolation (Fig. 7).

**Figure 7:**
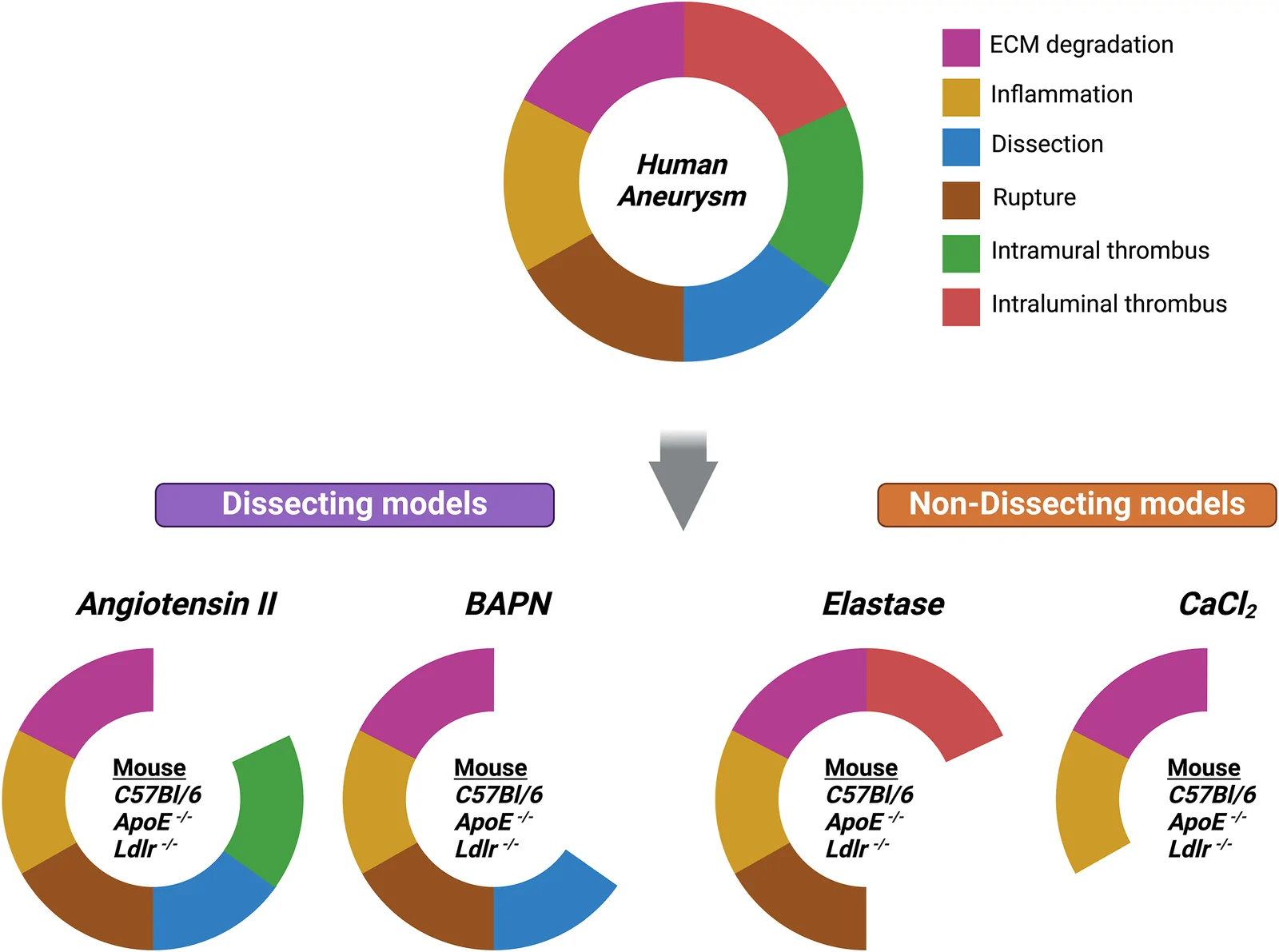
Integrative overview of murine models used to study aortic aneurysm and dissection. This schematic summarizes the major murine models of aortic disease and their ability to recapitulate key pathologic features observed in human aortopathy, including extracellular matrix (ECM) degradation, inflammation, dissection, rupture, intramural hematoma, and thrombus formation. Models are categorized into dissecting (Angiotensin II, β-aminopropionitrile [BAPN]) and non-dissecting (elastase, CaCl_2_) approaches. The **circular diagrams** provide a qualitative representation of the predominant pathologic processes associated with each model and do not reflect quantitative proportions. This integrative view highlights the complementary nature of murine models and their respective strengths in reproducing specific aspects of human aortic disease.

Rather than relying on a single experimental paradigm, the field is shifting increasingly toward use of integrative strategies that combine complementary systems. In this context, mouse models enable causal and mechanistic interrogation, bioengineered and patient-derived platforms capture human-specific biology, and computational approaches support multiscale prediction and data integration. The adoption of harmonized experimental standards, including the 2025 consensus guidelines, is essential to ensure rigour, reproducibility, and cross-study comparability.

Ultimately, progress in aortic disease research will depend on the precise alignment of experimental models with specific biological questions and clinical contexts. By integrating mechanistic insight with human relevance and standardized reporting, preclinical research can more effectively support the development of predictive diagnostics and targeted therapies.

Bridging experimental rigour with clinical relevance is essential to accelerating the translation of mechanistic insights into actionable strategies and advancing precision-based prevention of aortic disease.

## Ethics Statement

This article is a review of previously published literature and does not involve any new experiments, animal procedures, or the collection of biological samples. Therefore, ethical approval was not required.

## Patient consent

The authors confirm that patient consent is not applicable to this article, as this is a review based on previously published data.

## Funding Sources

This work was supported by Université Angers (University of Angers).

## Disclosures

The authors have no conflicts of interest to disclose.
